# Meanings of participation in a community initiative: a discourse analysis at Complejo SACUDE (Montevideo, Uruguay)

**DOI:** 10.3389/fsoc.2026.1881846

**Published:** 2026-07-24

**Authors:** Mayda Burjel-Verstraete, Manuel Montañés-Serrano, Iving Zelaya-Perdomo

**Affiliations:** 1 Faculty of Information and Communication, University of The Republic, Montevideo, Uruguay; 2 Department of Sociology and Social Work, University of Valladolid, Segovia, Spain; 3 Institute for Research in the Humanities, National Autonomous University of Honduras, Tegucigalpa, Honduras

**Keywords:** community initiatives, discourse analysis, participation, participatory spiral, public-community governance, Uruguay

## Abstract

Although the notion of participation pervades the literature on community initiatives, what those involved understand by it is rarely examined, even though such conceptions shape the methodologies adopted, the degrees of involvement enabled, and the coherence between intentions and practices. This article analyses the conceptions of participation held by those engaged in Complejo SACUDE, a public-community initiative co-managed by neighborhood representatives and municipal staff in the Casavalle basin, on the periphery of Montevideo, Uruguay. A qualitative-structural methodology was employed, based on the implementation of conversational devices distributed according to a structural sample designed to capture the social discourses associated with the relations relevant to the object of study. Four interrelated discursive positions were identified: committed participation; user participation; diverse participation; and progressive participation. The study concludes that participation is better represented as a spiral rather than a ladder: a nonlinear configuration in which eight steps feed back into one another and are not necessarily traversed in sequential order.

## 1 Introduction

A substantial body of scholarship addresses participation (Martínez-Palacios, 2018; Kaplún and Martínez, 2024; Bussu et al., 2022; Ahedo et al., 2023; Del Prado and Castelnuovo, 2026), and an equally extensive literature focuses on community initiatives (Gutiérrez, 2020; Rodríguez et al., 2023; Villasante and Burjel, 2026). Yet a question remains underexplored: what conception of participation is held by those who, in one way or another, take part in a community initiative? Addressing this question was the aim of the research on which this article is based. As Alzate (2020) and Cassetti et al. (2023) point out, the concept of participation is often taken for granted, even though its interpretations can vary widely; this makes it difficult to understand what people mean by “participating” in specific contexts. This insight is crucial given that the conceptions and perceptions of those involved in the design and implementation of participatory processes influence the methodologies adopted, the degree of engagement among different stakeholders, and the alignment between stated intentions and actual practice (Mariani et al., 2025).

To address this need, the research analyzed the discourses of those participating in a community initiative known as the Complejo SACUDE (henceforth SACUDE). The initiative is located in the Municipal neighborhood, within the Casavalle basin in Municipality D, on the periphery of Montevideo, Uruguay, and is jointly managed by the city government and neighborhood representatives. It can be characterized as a collaborative governance initiative that emerged in 2010 through grassroots mobilization (Blas and Ibarra, 2006)—that is, from the bottom up—when organized residents demanded the modernization of facilities and the expansion of services offered to the public in a space that had originally served as the meeting hall of the neighborhood’s Pro-Development Commission. The acronym SACUDE refers to the initiative’s three core areas: salud, cultura and deporte (health, culture, and sports). The facility comprises a polyclinic, a theater, a gym, an amphitheater, two sports fields, and rooms hosting more than 50 cultural workshops, sports activities, and health promotion programs each year, alongside additional events such as film screenings and theater performances. The vast majority of activities are offered free of charge. Annual workshops register an average of 1,500 participants, and summer activities draw an average of 700 participants. Within the Montevideo City Government, the initiative falls under the Cultural Decentralization Division of the Department of Culture.

SACUDE is the outcome of organized neighborhood action, which not only demanded quality social infrastructure in a long-neglected area of Montevideo but also sought a role in its management. This form of shared management situates the initiative within the realm of democratic innovations (Elstub and Escobar, 2019) and, more specifically, among experiences of collaborative public–community governance currently being replicated at the local level in diverse settings (Gorostidi and Martínez, 2023; Telleria and Ahedo, 2015; Díaz et al., 2021). The initiative’s stated mission is to promote community participation through a co-managed project grounded in a rights-based and socially equitable approach, as a means of individual and collective transformation for the residents of the Casavalle basin, Municipality D, and the city of Montevideo (Complejo SACUDE, 2026). SACUDE is governed by a Co-Management Commission composed of technical staff assigned by the Montevideo City Government—a management coordinator and area coordinators for health, culture, and sports—together with neighborhood representatives, one per area, elected by vote.

Within this context, this work examines how participation is conceived by those involved in SACUDE, considering the meanings attributed to participation in a community initiative. By analyzing these discourses, the article seeks to contribute to current debates on participation and community initiatives, particularly by exploring how participation is conceived, practiced, and interpreted by those who sustain such processes.

## 2 Literature review

Understanding what participation means within an initiative such as SACUDE first requires situating the term within the broader spectrum of practices it encompasses. People participate at different times, in different settings, and in different ways, with varying degrees of intensity: voting, joining a protest, taking part in a performance, attending a dance workshop, playing on a soccer team, or sitting on a neighborhood committee, among many other forms. These are instances of participation experienced on an everyday basis, though with markedly different implications. Participation permeates social life and operates simultaneously as a human need and as a means of meeting other needs, a dual condition that underpins the case for convivial models of need attention, in which the synergistic satisfaction of human needs is sustained through community-based arrangements (Ramos, 2021). The meanings that people attribute to the act of participating are, in fact, manifold and depend on their life trajectories, social positions, and the contexts in which they engage, giving rise to a plurality of participatory subdomains that no one-dimensional framework can fully capture (de Wind et al., 2019).

Max-Neef et al. (1986) consider participation to be one of the nine fundamental needs and distinguish between needs and satisfiers, the latter being understood as the ways of doing, being, having and interacting that enable these needs to be met. In their words, “what is culturally determined is not human needs but the satisfiers of those needs” (p. 27). From this perspective, participation constitutes not only a need but also a “synergistic satisfier” (Max-Neef et al., 1986), insofar as the satisfaction of one need simultaneously contributes to the satisfaction of others. Díaz Bordenave (1989) also refers to participation as a need and as a means to satisfy needs, distinguishing between basic needs (such as eating, drinking, sleeping, working, and receiving an education) and needs that are “not obvious but equally powerful, to the point that their unmet state causes severe damage to the personality” (Díaz Bordenave, 1989, p. 19). Among these, he includes thinking freely, expressing oneself freely, belonging to a group, being recognized as a unique, valued, and respected person, creating and recreating a physical and cultural environment, and having a say in decisions that affect everyone. All these “non-obvious” needs, he asserts, can be met through participation. The recognition of participation as a need has further led to its consideration as a right, enshrined in various international instruments that either recognize it explicitly or imply it.

Participation is, therefore, a human need, a means of addressing other needs, and, at the same time, a fundamental right. Yet, as already noted, participating in a parents’ meeting is not equivalent to taking part in a protest, joining a neighborhood group, or attending a public consultation. It is therefore necessary to distinguish among the various forms that participation can take. Some authors identify levels of participation or different participatory states or roles (Plaza, 2007, p. 135). Arango (2007) identifies several forms of participation: spontaneous participation, which arises organically without stable organization or clearly defined purposes; imposed participation, in which people are compelled to undertake actions or join groups; voluntary participation, in which a group driven by its own members defines its organization, methods, and objectives; instrumental participation, manipulated by external agents; and granted participation, in which certain groups are allowed to influence planning within a specific project.

**Figure 1:**
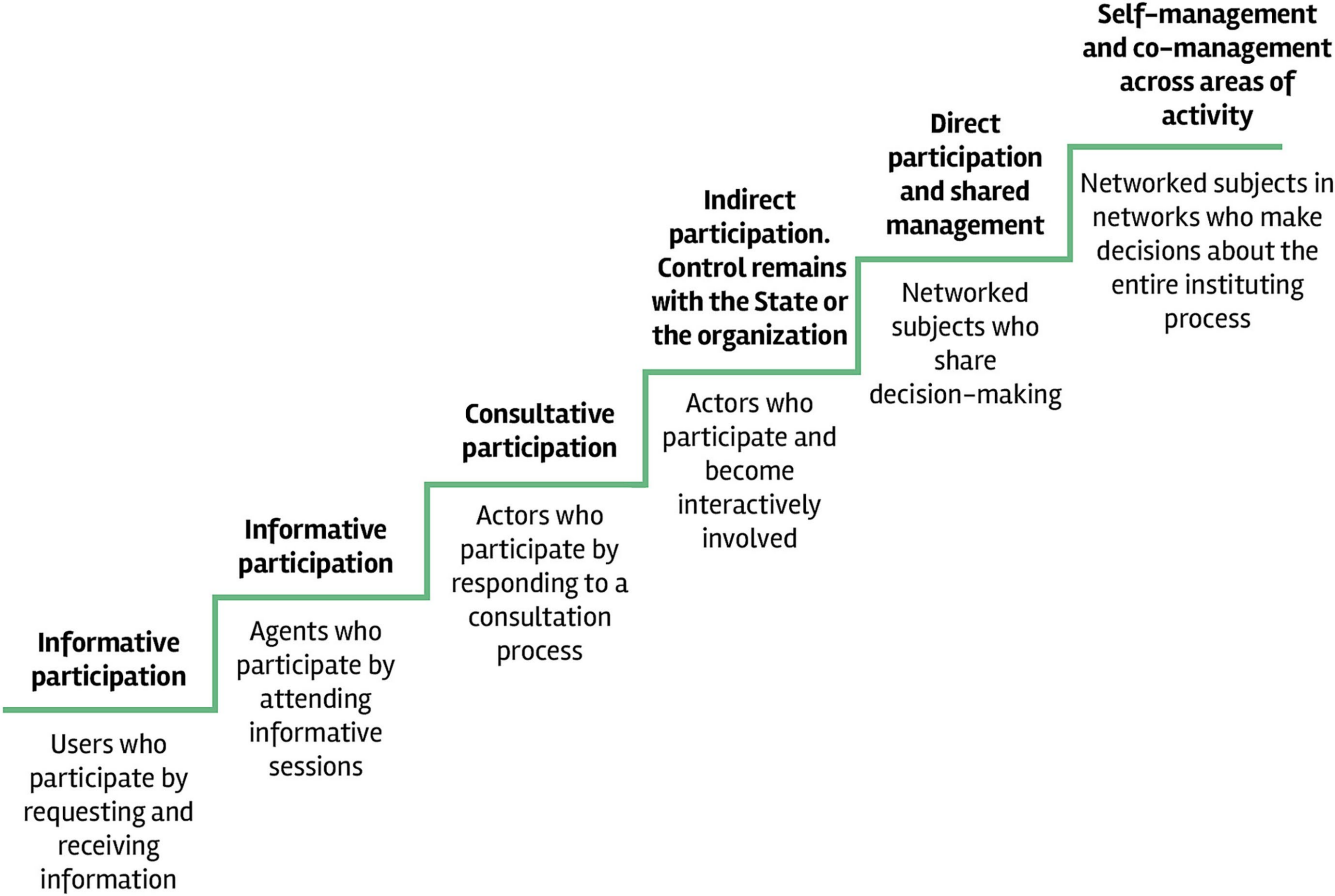
Ladder of participation. Adapted from Red Cimas (2015).

The professionals who constitute Red Cimas (2015) draw on Arnstein (1969) and Hart (1993) to propose a ladder of participation. As shown in Figure 1, the lower rungs of this ladder correspond to participatory processes associated with merely being informed; the middle rungs comprise instances of consultation without decision-making; and the upper rungs include co-managed and self-managed participatory processes. Movement up the ladder is understood as movement toward participatory democracy.

Some scholars further subdivide these categories. Díaz Bordenave (1985) distinguishes, within the consultation level, between optional consultation—in which an organization’s leaders request criticism, suggestions, or data from members in order to address problems—and mandatory consultation, in which members must be consulted but the final decision still rests with the leadership or institution. The same author identifies three sub-levels within the participatory level of management: co-management, delegation, and self-management. The latter is identified as the highest form of participation, in which the group sets its objectives, chooses its means, and establishes the relevant controls without reference to any external authority. In a related vein, Blas and Ibarra (2006) note that, depending on its function, participation may be informative (bottom-up or top-down), consultative (mandatory or voluntary), co-decisional, decisional, or co-managerial.

Regarding the relationship between participation and the State, Blas and Ibarra (2006) distinguish between participation by invitation and participation by intrusion. In the former, public institutions—governments, municipalities, and the like—invite citizens to take part in various processes and spaces. In the latter, citizens or social organizations enter the public sphere on their own initiative and demand recognition from the administration. Sirvent (1984), in turn, distinguishes between real participation, located on the upper rungs of the ladder, and symbolic participation. Real participation, in this view, exists when the members of an institution or group exercise power across all processes—through decision-making at its various levels, the implementation of those decisions, and the ongoing evaluation of institutional functioning. By contrast, symbolic participation refers to processes tied to actions that exert minimal influence or that generate, in individuals or groups, the illusion of exercising a power that does not actually exist.

The gap between declared participation and actual participation has been empirically documented in recent studies of community health projects, where, despite self-descriptions as highly participatory, the predominant forms barely move beyond attendance at activities or occasional consultation, with very few examples of joint decision-making (Cassetti et al., 2023). The discrepancy, the authors warn, is linked to the absence of an institutional culture of participation, the rigidity of available regulatory frameworks, and the lack of specific training for those who promote such processes. They go so far as to suggest replacing the term “participate”—too often used as a synonym for “attend”—with expressions that more accurately reflect actual degrees of involvement, such as “consult” or “engage.” Along similar lines, López-Ruiz et al. (2024) observe that the actions prioritized by community projects tend to concentrate on organizational and communicative improvements, while more structural dimensions—diversity within the steering group, evaluation of outcomes, work–life balance measures, and the inclusion of vulnerable groups—are sidelined. This finding underscores that profound transformations in participation require sustained timeframes and dedicated resources that are rarely available.

Recent literature also challenges the very metaphor of the ladder, criticizing its linear, sequential, and unidirectional character, which misleadingly suggests that greater inclusion automatically translates into greater influence (Den Dulk and Buizer, 2026; Koomson, 2024; Rosen and Painter, 2019). In contrast to this model, Rosen and Painter (2019) propose conceptualizing participation as a dynamic and iterative process of co-production, in which community actors are not only invited to make decisions but also provided with the resources, capacities, and institutional support needed to sustain their influence throughout the entire project cycle. Co-production, they argue, is neither a destination nor a fixed stage, but a horizon under permanent construction that requires the continuous dismantling of the power and resource asymmetries that limit community control, even when such control has been formally recognized. In a complementary direction, Koomson (2024) proposes adopting “involvement” as a unifying category and understanding it as a bidirectional continuum, in which both the willingness of external promoters to cede spaces of power and the efforts of local participants to occupy those spaces and exercise real influence over decisions reinforce one another. In this model, the weaker forms—pseudo-involvement and partial involvement—do not constitute fixed steps but rather moments within a process that may progress, stagnate, or regress depending on context. Den Dulk and Buizer (2026), for their part, suggest using the plural form “participations” to account for the multiple forms—formal and informal—in which people are included in or excluded from community spaces, forms that coexist and often contradict one another within the same territory.

Achieving genuine participation can therefore be understood as a learning process that involves traversing intermediate, semi-participatory steps, in which the conscious awareness of those involved is essential. This understanding aligns with viewing participation as a process of critical reflection on reality that encompasses all those involved, and one that can only unfold to the extent that it is sustained by a problematizing dialogue capable of fostering reflection rather than merely soliciting opinions, as if those consulted were the sole holders of the truth. The matter is not merely the impact of opinions or whether they are binding; rather, real participation entails fostering a critical disposition and reflecting on starting points, which presupposes an educational process grounded in dialogic relationship, in the terms proposed by Freire (1993): “Participation as an exercise of the voice, of having a voice, of intervening, of deciding at certain levels of power, as a right of citizenship, is in direct and necessary relation to progressive educational practice” (p. 82).

Building on this concern, Villasante et al. (2012) argue that simply creating spaces for conversation is not sufficient, since residents are themselves shaped by the dominant frameworks of meaning through which they interpret reality. Similarly, Montero (2005) observes that participation does not occur in isolation from the practices, beliefs, and values prevailing in the local context in which people live; certain ways of life and cultural patterns present in a community may contribute to reproducing exclusion, mistreatment, risk, or ignorance regarding particular phenomena. Any dialogic process aimed at fostering participation must therefore promote critical reflection on the frameworks through which each person perceives social reality (Villasante et al., 2012). The task is one of observing and reflecting on reality in order to transform it. This demand for reflexivity also extends to those who facilitate such processes: contemporary feminist and participatory practice insists that researchers and promoters must critically examine their own positionality, their affects, and their place within the power relations that traverse community spaces, rather than presenting themselves as neutral agents (Alzate, 2020).

These conditions, which affect all participants, bring power into play in its many forms, making it essential to ask, in every participatory process, whom it serves. Brandão (1985) argues that the meaning of participation can only be fully grasped by examining the type of power it serves and the project of development or transformation with which it is articulated; from this perspective, valuing popular participation and defining its strategy require thinking about it in relation to the social production of power by popular sectors. In a similar vein, Giorgi et al. (1995) warn that participation is not a neutral concept but an ideologically charged notion whose meaning depends on the structure and intent of the proposal in which it is embedded. It is also necessary to consider that power struggles are an inherent dimension of all human relationships and unfold on every scale. Participation should not be romanticized: such struggles are present in every organization and collective, whether a political party, a neighborhood group, a community development committee, or a family. Recognizing this, however, does not entail concluding that every form of power is oppressive. Montero (2005) points out that, even within everyday life, there exist expressions of power that make possible the changes demanded by various social actors. Distinguishing oppressive forms of power from those that are not, is part of the critical reflection that processes of deep participation entail.

The participation under discussion here, with its various features, forms, and levels, is often qualified as social, civic, or community participation. Although these notions are frequently used as synonyms, they are not interchangeable: each carries distinct features. In broad terms, social participation encompasses both civic and community participation, insofar as all participation unfolds within social and historical contexts that simultaneously shape both the individuals and the groups in which it takes place (Ussher, 2008). Although there are differing conceptions of social participation, it is generally understood as the capacity to influence or make decisions on issues that affect people, whether by voting, joining a neighborhood group or social organization, or taking part in a protest. Civic participation, in turn, refers to the involvement of individuals in the public sphere—both state and non-state—in their capacity as members of a political community. To that extent, it is closely tied to the model of democracy and the type of relationship between government and society that one seeks to build (Villarreal, 2009).

Community participation—the concept embraced by SACUDE in its mission—falls within both of these approaches but acquires its distinct character in the community sphere. Montero (2005, p. 107) understands it as “an organized, collective, free, and inclusive process involving a variety of actors, activities, and levels of commitment, guided by shared values and objectives, the achievement of which leads to community and individual transformations.” Recent research on how those who coordinate participatory initiatives conceive of participation converges in describing it as an act that is at once political and relational, involving shared responsibility, information exchange, empowerment, and commitment to the common good—even as it coexists with the risk of being reduced to empty rhetoric or mere institutional formality (Mariani et al., 2025). As Ahedo and Ibarra (2007) argue, participation entails sharing power, and shared power requires a demos—an “us”—that is built within the community sphere. From this perspective, the endurance of democracy depends on both the construction of collective subjects that nourish and reinforce community infrastructure and the existence of mechanisms for deepening democratic life (Ahedo et al., 2023, p. 43). Accordingly, participation is defined as a process of community organization or mobilization through which people consciously recognize and assume their role as agents of collective becoming, constituting both a personal practice and a collective process oriented—or potentially oriented—toward formation, education, creativity, equality, and transformation (Ahedo, 2022).

Reflecting on the common can mistakenly lead to the assumption of homogeneous groups grounded in a shared egalitarian identity. It is therefore important to emphasize that community, as the term is reclaimed here, does not rest on sameness but on the recognition of what is held in common across multiplicity. In this sense, it fosters the coexistence of “the one and the multiple” (Plaza, 2007; Rivera Cusicanqui, 2018). This multiplicity refers both to the articulation of collective and individual identities and, in territorially rooted communities, to the very physical spaces through which community life is lived and constituted. The reference here is to spaces in which meanings converge: interstitial spaces with diffuse boundaries (Álvarez Pedrosian, 2018; Álvarez Pedrosian et al., 2019); to territories in the plural (Álvarez et al., 2021); or to territorialities (Álvarez Pedrosian, 2018), understood as places invested with diverse meanings by those who inhabit them (Álvarez Pedrosian and Blanco Latierro, 2013). For Villasante (2006), community cannot be understood as a fixed unit of identity awaiting recovery. Rather, it is a space traversed by internal and external conflicts, in which processes of identification take shape through both formal and informal relational networks, and in which the affective dimension of the group is as significant as its socioeconomic structure and the ideologies at stake.

The common is therefore reclaimed here not as something essential, but as something continuously constructed and reconstructed within and through multiplicity, enabling the reproduction of life in everyday practices. Segato (2018) contrasts the political project of bonds with that of things, arguing that the former fosters reciprocity and produces community, whereas the latter serves capital and produces individuals. This perspective underpins the case for community-based work, grounded in the conviction that it is through bonds—through the proximities of micropolitics—that processes of critical reflection can emerge, making possible desired social transformations and contributing to the “good living” of the majority. In examining the community dimension of the Complejo SACUDE, the focus is placed on interactions among people—and networks—in which bonds of proximity predominate, in a specific socio-territorial sphere that extends beyond neighborhood boundaries or any other administrative division. These bonds involve emotions, conflicts, and collective memories, and they shape the meanings attached to participation in a community initiative. The analysis draws on Montañés (2022) to approach meaning as both interpretive and affective: it refers, on the one hand, to the concrete sense people assign to something in a specific situation and, on the other, to the feelings through which that situation is experienced. In this view, understandings of the world—and therefore of participation—are always both practical and emotional.

## 3 Materials and methods

To gather the discursive raw material through which the concept of participation could be examined, the study employed conversational devices (CD): moderated group discussion sessions organized in accordance with the sample described below. The term device is used here to refer to a tool for generating meanings related to participation—meanings shaped by the experiences of those involved and by the networks in which they are embedded, as expressed through their discourses. For Foucault (1994), the device is precisely the fabric woven among diverse elements, encompassing both the said and the unsaid. The *dispositif* emerges at a particular moment, fulfills a strategic function, and is traversed by relations of power and knowledge (Agamben, 2011). In this study, the conversational devices functioned as methodological constructs: collective spaces with distinct characteristics. The meetings were organized according to the structural sample previously designed, so that the composition of each meeting was homogeneous. This made it possible to avoid potential relations of power, superiority, or real or symbolic domination. At the same time, each meeting incorporated the corresponding inclusive heterogeneity which, without disrupting the substantive character of the group, could generate debate. In each case, an atmosphere of trust was fostered in order to encourage spontaneous conversation among people who already shared some degree of familiarity, while also seeking to avoid the reproduction of asymmetrical power relations. To this end, participants were told that there were no better or worse opinions, nor opinions that were more or less relevant, but rather that all inputs were valid. All contributions were anonymized in the transcripts and in the reporting process to protect participants’ privacy. Moderators—members of the steering group who also belonged to the different clusters represented in the sample—were instructed to facilitate discussion not through a question-and-answer format, but in such a way that, after the introduction of a discursive prompt, opinions, questions, and debates would emerge; in short, a dense discursive texture.

To capture the range of discourses produced, a structural sample was employed. In this type of sampling, representativeness is grounded in the principle of socio-structural representativeness, understood as a methodological tool for bringing social discourses to light (Mejía, 2000). It is determined by “the saturation that occurs when all possible discourses related to the research problem are recorded” (Montañés, 2013, p. 842). Saturation and representativeness are thus achieved by including all the groups that, through their discourses, reproduce the relations relevant to the object of study (Montañés and Lay, 2019). Discursive positions, in this framework, are “socially defined typical discursive roles that subjects adopt in their specific discursive practices” (Ruiz, 2009, p. 6). These positions bring together discursive contents whose repetition and internal consistency justify their configuration as stances—distinct from, opposed to, different from, or akin to others—within a relational structure (Lay and Montañés, 2013).

**Figure 2:**
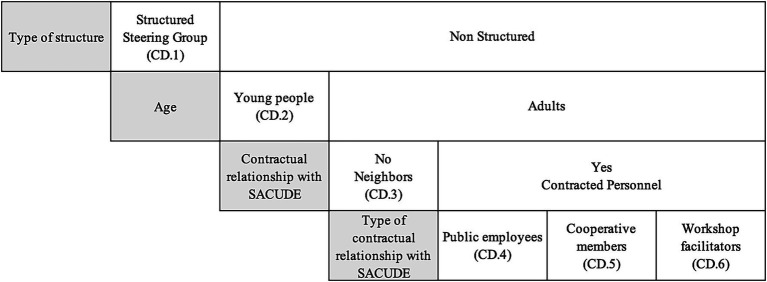
Graphical representation of the structural sample.

In designing the sample, the relevance of particular networks is assessed through the selection of structuring axes. These axes define the thematic field, the temporal frame, and the socioeconomic and cultural characteristics of the population that shape the conversational practices through which discursive material is produced and from which discursive positions can subsequently be inferred (Montañés, 2013). The resulting discourses reveal the different ideological positions attributed to a shared social reality. Ideology is understood here as “the ideas and concepts that structure thought in a coherent and structural manner” (Montañés, 2013, p. 842), guiding the meanings inferred from what is said and which discourse analysis seeks to bring to the surface. As shown in Figure 2, the structural sample was designed based on information gathered in both the meetings of the Steering Group and those of the SACUDE Co-Management Commission, which served as the Research Monitoring Commission. In accordance with this sample, different conversational devices were used to record the discursive raw material provided by the group categories identified within it. In accordance with the sample, six conversational devices were used between August and December 2022, each involving between eight and ten participants. The number of participants in each group conversation was kept below ten in order to enable dialogue and mutual questioning. In total, 58 people participated in these meetings, with a similar proportion of men and women. At the outset, the purpose of the activity was made explicit: the aim was not to reach group conclusions but to foster open conversation without concern for finding the right words. The prompts were as follows: *What is meant by participation? and How would you define participation?*

It might be objected that the sample lacks certain group configurations if the variables are cross-tabulated in the manner of a stratified statistical sample. In a structural sample, however, the aim is not to mechanically cross-tabulate all meetings or all categories derived from the axes considered relevant. The structural/qualitative sample should not be confused with a stratified statistical sample: it is governed neither by statistical criteria nor by a mechanical juxtaposition of social structures. Its purpose is not to reflect discourses associated with specific socio-statistical categories but to capture those that emerge from the relations defined by the structural axes. The sample is thus designed to generate discursive raw material that enables the analysis of the social narratives linked to those relations, rather than to represent sociocultural aggregates of individuals. Accordingly, each meeting included in the structural sample is not, in itself, necessarily representative of the category to which it refers; rather, it is the joint analysis of the discursive material produced across all groups that provides a comprehensive view of the discourses associated with the relations defined by the structuring axes. It is in this sense that representativeness was achieved. As noted above, representativeness is attained once discursive saturation is reached, that is, when the data collected are sufficient to capture all the discourses relevant to the purpose and objectives of the study.

According to Montañés and Lay (2019), once the discursive raw material produced in the conversational settings has been transcribed, two paths are available for its interpretation: analyzing the text produced in each group setting separately, or analyzing the discursive fragments extracted from each group in relation to the thematic blocks defined for the research. The second option was selected, since the aim was to bring out the different discursive positions regarding the object of study, namely participation. The discourse analysis was carried out in successive stages. In the first stage, coding, codes were established in relation to the thematic blocks, and textual fragments alluding to those blocks were selected. These codes served as the starting point, as subsequent readings allowed additional codes to be defined and assigned according to their relevance to the object of study. The process was carried out using Atlas.ti software, which made it possible to export reports grouping textual fragments by code and thus facilitated the stage of categorization, namely the construction of new analytical texts organized according to thematic blocks. Some fragments were assigned to more than one code, in which case they appeared in multiple reports.

In the phase of discursive inference, the new texts were analyzed and categorized by thematic block. An initial interpretation was developed through tables in which textual fragments associated with the same code were grouped in one column, while the corresponding analysis was presented in another. Within each row, fragments were grouped by affinity so that they could be interpreted together. Following further readings, the relational phase sought to define the discursive positions, establish their conceptual definitions, and ultimately construct a graphical representation illustrating the relations among them.

## 4 Findings

The discursive positions identified through the analysis of the discourses on how those involved in a community initiative conceptualize participation revolve around social commitment. Social commitment is understood here as a sense of belonging to something—a project, a group—together with a willingness to act for the common good, in ways that transcend individual well-being. From this perspective, participation entails dedication, responsibility, and the capacity to make decisions.

Participating is commitment; it’s not just coming to watch a movie or a play and that’s it; it’s getting involved… It’s also responsibility… It’s time and willingness… It’s all of that together. It’s making decisions, participating in the community… it’s feeling like you belong… and commitment is getting involved with others for the sake of something. (CD.2)

This stance is distinguished from merely attending an activity, since commitment, in this view, entails more than simply showing up.

They come eager to be in a workshop—that’s what it means to me—but being part of a workshop and doing an activity makes me wonder whether they’re actually participating or not […] Maybe that’s the difference between participating and attending […] people who, besides, have every right not to want to be part of it […] For me, among many other things, it means getting involved with commitment in something that is shared and for the benefit and service of that community. It can also mean participating in other things, but I think it’s about commitment and being committed. (CD.3)

**Figure 3:**
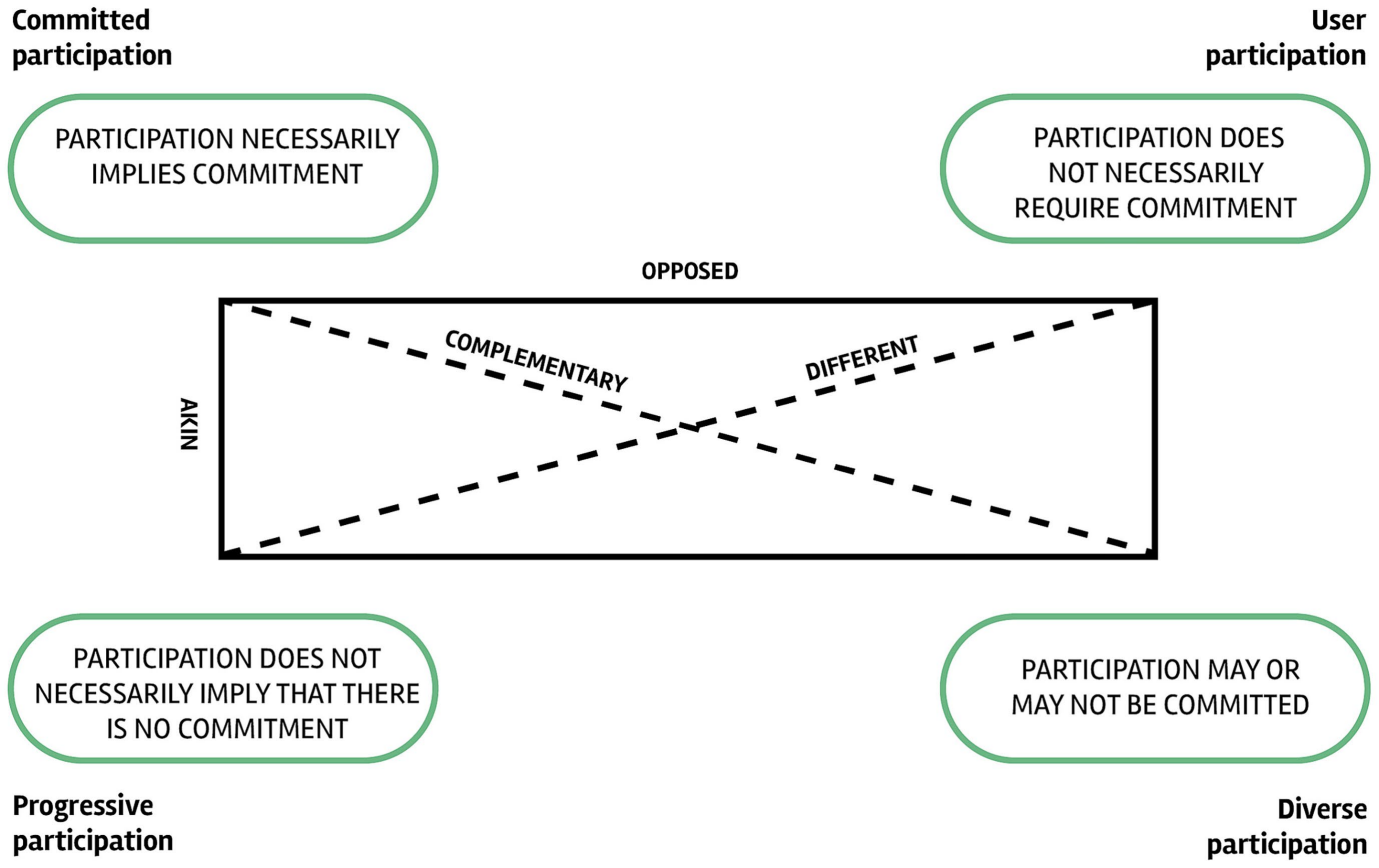
Relations among discursive positions on conceptions of participation.

On the basis of these discourses, two discursive positions can initially be distinguished: the committed position and the position that holds that participation need not necessarily entail commitment. The sociological application of the semiotic square developed by Greimas and Courtés (1990, 1991) yields, however, a more nuanced picture. As Figure 3 illustrates, not two but four interrelated positions emerge, defined by relations of discursive opposition, difference, and affinity. In addition to the two opposing positions already identified, a third position—distinct from the first—can be recognized when it is assumed that participation may or may not involve commitment, and a fourth—akin to the first—when participation is understood as compatible with commitment, even if commitment is not taken to be a necessary condition.

The semiotic square is applied here in accordance with the sociological approach to discourse analysis (Conde, 2009), since what must be avoided is a mechanical articulation of a linguistic structure devoid of empirical grounding or of concrete reference to the social reality under study. As Abril (1999) cautions, the semiotic square is a key operational component within its semi-narrative model, but outside that methodological context it risks becoming a trivial exercise.

Drawing on the analysis of the discursive raw material produced across the various conversational devices employed in the study, four discursive positions were identified. These are: committed participation (A), which defines commitment as an essential condition for participation to exist; user participation (B), discursively opposed to the first, which holds that participation does not necessarily require commitment; diverse participation (C), distinct from the first, which maintains that there is no single way of participating, but rather a plurality of forms; and progressive participation (D), akin to the first, which holds that participation does not necessarily imply the absence of commitment. From this discursive position, participation is understood as a process through which one may initially engage in an activity without social commitment and, progressively, develop an interest in issues related to the planning, management, and decision-making processes that affect the community initiative.

The empirical legitimacy of the first position, (A) committed participation, has been substantiated in the preceding paragraphs. Position (B), user participation, holds that participation does not necessarily entail commitment: one participates insofar as one attends or engages in an activity as a user.

But you can participate here at SACUDE when you come to the theater, and you come… When you go to a workshop, an event, or other activities, simply as users… or when you play sports. (CD.3). It’s the same when we talk about neighbors and all that; once they walk through the door, they’re already participating in the activity and everything. (CD.4)

Position (C), diverse participation, holds that participation may take both committed and non-committed forms. It recognizes a plurality of modes of participation and varying degrees of commitment, and explicitly invokes the notion of levels of participation. Some discourses refer to this diversity as a whole, while others refer to only one of its modalities, such as participation through expressing opinions, receiving information, or providing it.

How and when one participates, in any form, at any time—there is no single moment or way to participate. (CD.5). Here, some people participate by attending a workshop, as users of an educational or cultural proposal. And there are people who come to participate by suggesting ways to improve the SACUDE program, and there are different levels of participation. […] I found myself thinking about the levels of participation at the polyclinic, and one thing is simply coming to get care, another is coming to get care and recommending someone else to come, and yet another is when the community health worker tells you, “Let’s prioritize this health issue because I have that neighbor who doesn’t come to the doctor…” […] There are different levels of feeling like you belong and of empowerment within the healthcare setting. (CD.1)

Position (D), progressive participation, holds that participation does not necessarily imply the absence of commitment. Rather, the discourses point to a form of commitment that develops progressively over time and may begin with attendance at an activity. Participation in a workshop, for example, may constitute a starting point of a process that gradually leads to involvement in actions shaped by social commitment.

I think about it more from the very outset. All of us are here to generate participation. All the workshops we run, all the sports activities, the entire range of activities on offer is designed so that people participate, or at least begin to connect, from the very start, with the complex [SACUDE]. From what happens in those practices, a person will then either continue to get involved or not […] Someone may begin by taking part in a workshop, then helping the workshop facilitator, lending a hand in another workshop, or joining a committee in order to give participation some thought and direction […] The most basic level, it seems to me, is to invite them to be part of the workshop; once you have a base of participants, then the aim is to make them active participants. (CD.6)

Within this process—which, as noted, may begin with the simple act of attending an event—participation unfolds through the successive steps of a participatory spiral. From this perspective, meaningful participation entails engagement with each of these steps. The first step is constituted by the seeking, sharing, and circulation of information.

What happens here is conveyed outward. We, the ones who participate, are the recipients, and we have to pass it on, take what is said here to the community, to the neighborhood, to the people, so that people know. (CD.3)

A second step emerges when, beyond gathering and transmitting information, discussion is fostered and opportunities are created for the expression of opinions.

But in the Sports Commission, for example, everyone discusses the problems or the things that could be improved. It does not necessarily have to be a problem; it can also be something that is already good and could still be improved. (CD.2) At a certain point, there are things that those of us participating in different spaces begin to question, for example, why young people were only allowed to organize dances and not be involved in management… windows began to open (CD.1). Participation in the health assemblies—is that not a milestone? They were valuable because they involved the neighbors. The first assembly of Municipality D was in 2010. It was the neighbors coming together to exchange views on the health problems they identified in their neighborhoods […] When you give an opinion in order to contribute something. That is it, that is when you are participating through your opinion. (CD.4) And because I come, I give my opinion, what I think, what I like, what I do not like […] always with respect, without attacking the other male or female participants. (CD.2)

A third step concerns the need to foster both individual and collective reflection so as to cultivate a critical disposition capable of challenging taken-for-granted assumptions. As the classical tradition has long warned, the dominant ideology is the ideology of the ruling class.

Participation implies precisely that: opening minds. (CD.3). We define it and subscribe to the concept, but behind that there is an ongoing process of construction and critical analysis so that, when situations or barriers to participation arise, alternative strategies can be developed (CD.4)

The fourth step points to the collective and participatory construction of knowledge. Participation, it is understood, requires the recognition of disagreements and the development of proposals that go beyond any single individual’s initial ideas.

We may find ourselves in a space where an idea is put forward that is not the one I had in mind. An idea may begin as A and end up as Z, and we build it together, but that process is not always rosy. Along the way, things happen to us as a group, do they not? Because there is always that feeling that I want it to be my idea and, well, the other person has to wait a little, has to see how that idea gradually takes shape. (CD.6)

A fifth step fosters proactive participation. It is argued that the collective and participatory construction of knowledge must be articulated with the collective formulation of proposals. Participation, in this sense, involves devising plans, programs, and actions for the well-being of the community.

When you want to build something, right? You participate when you want to build something too. Here, people want to build a lot of things; there are many goals. (CD.5). Participation involves an activity of construction and, precisely, a sense of being part of something in order to make it different, to move beyond complaining and get things moving. (CD.4). Reference is made to construction as the projection of an action for the benefit of the community. To solve things and create, you can build things by participating. (CD.3)

The sixth step occurs when, following discussion, reflection, and planning, participants are empowered to take part in decision-making.

The first two things [participation] means are that it is a right and that it also involves decision-making by the community […] For me, what ultimately defines something as participatory has to do with the possibility of taking decisions […] That neighbor who also comes with the intention of taking part in decisions regarding the concrete processes taking place in SACUDE. (CD.1)

The seventh step concerns participation in the implementation of the proposals adopted. In this view, it is through involvement in their execution, that people come to feel genuinely part of a collective project.

It is much richer when things are done among several people, together with institutions, than when ideas or projects simply come down and one can only take part in basic ways. For example, you might have a dance, and I can come and dance, but it is not the same as if we organize it, or if you come to a party because you are invited as opposed to putting the party together with others. It has a different feel to it, a different kind of enjoyment. It lies in getting involved and enjoying that. (CD.3)

The eighth step refers to participatory evaluation. Evaluating both the process and the steps taken should serve not only to improve what has already been accomplished, but also to guide the formulation of new initiatives for the benefit of the community.

In the committee, everything that is happening at SACUDE is evaluated, and we see what can be improved or not and what things there are to change. […] There, in the committees, everyone kind of speaks their mind, and many things are evaluated. (CD.2)

## 5 Discussion

From the perspective of position (D), progressive participation, committed participation and user participation are best understood as two dimensions of participation that should be fostered simultaneously. This entails recognizing the transformative potential of access to the cultural, sports, and health promotion activities offered by the initiative while, at the same time, pursuing committed participation as SACUDE’s overarching goal. This duality resonates with recent findings indicating that the act of participating is experienced as a multidimensional reality in which heterogeneous subdomains coexist, ranging from everyday sociability to civic engagement, and whose significance depends on each person’s life trajectory, social position, and circumstances (de Wind et al., 2019). Embracing this heterogeneity means refraining from organizing forms of participation into a single hierarchy and recognizing instead that different modalities may be equally legitimate and valuable, depending on the stage of the personal and collective process in which they occur.

A further consideration is that committed participation cannot be reduced to ensuring that people take part in SACUDE’s decision-making or planning bodies—namely the Co-Management Commission and its thematic subcommittees. Commitment to social engagement oriented toward the well-being of the majority can take many forms and should ideally extend beyond the initiative itself. SACUDE may aspire to become a project increasingly shaped by committed participants; however, fostering participation also requires conceiving it as something that exceeds formalized organizational spaces. Care must therefore be taken not to channel users exclusively into the organization and its predefined forms of engagement. This caution is consistent with critiques in the contemporary literature of processes that, under the rhetoric of inclusion, confine participation within rigid institutional frameworks and thereby displace the participatory practices already present in local communities (Den Dulk and Buizer, 2026). Referring to participations in the plural makes it possible to recognize that, within the same environment, multiple ways of engaging in the collective project—both formal and informal—coexist, and that reducing them to institutionally prescribed channels risks silencing others that are equally significant to the community.

Promoting socially committed participation and fostering committed participation within SACUDE are therefore two objectives that may converge, yet remain distinct. Achieving both requires the design and implementation of strategies that encourage committed participation while simultaneously positioning SACUDE as a space in which such participation can be exercised. The aim is to inspire those who engage with the initiative and to create experiences through which they may become informed, express opinions, debate, reflect, decide, and take part in the design, implementation, and evaluation of proposals. This dual horizon aligns with what recent research has identified as a recurring pattern in community initiatives: even when those driving them understand participation as a political and relational act with transformative potential, in practice efforts tend to concentrate on organizational and communicative improvements, while the more demanding dimensions—such as shared evaluation, diversity within the steering group, and effective redistribution of power—are often sidelined due to insufficient time, resources, or institutional culture (López-Ruiz et al., 2024; Mariani et al., 2025). Acknowledging this tension does not mean abandoning the goal of commitment, but rather recognizing that its realization requires long-term sustainability and deliberate attention to the material and institutional conditions that make it possible.

**Figure 4:**
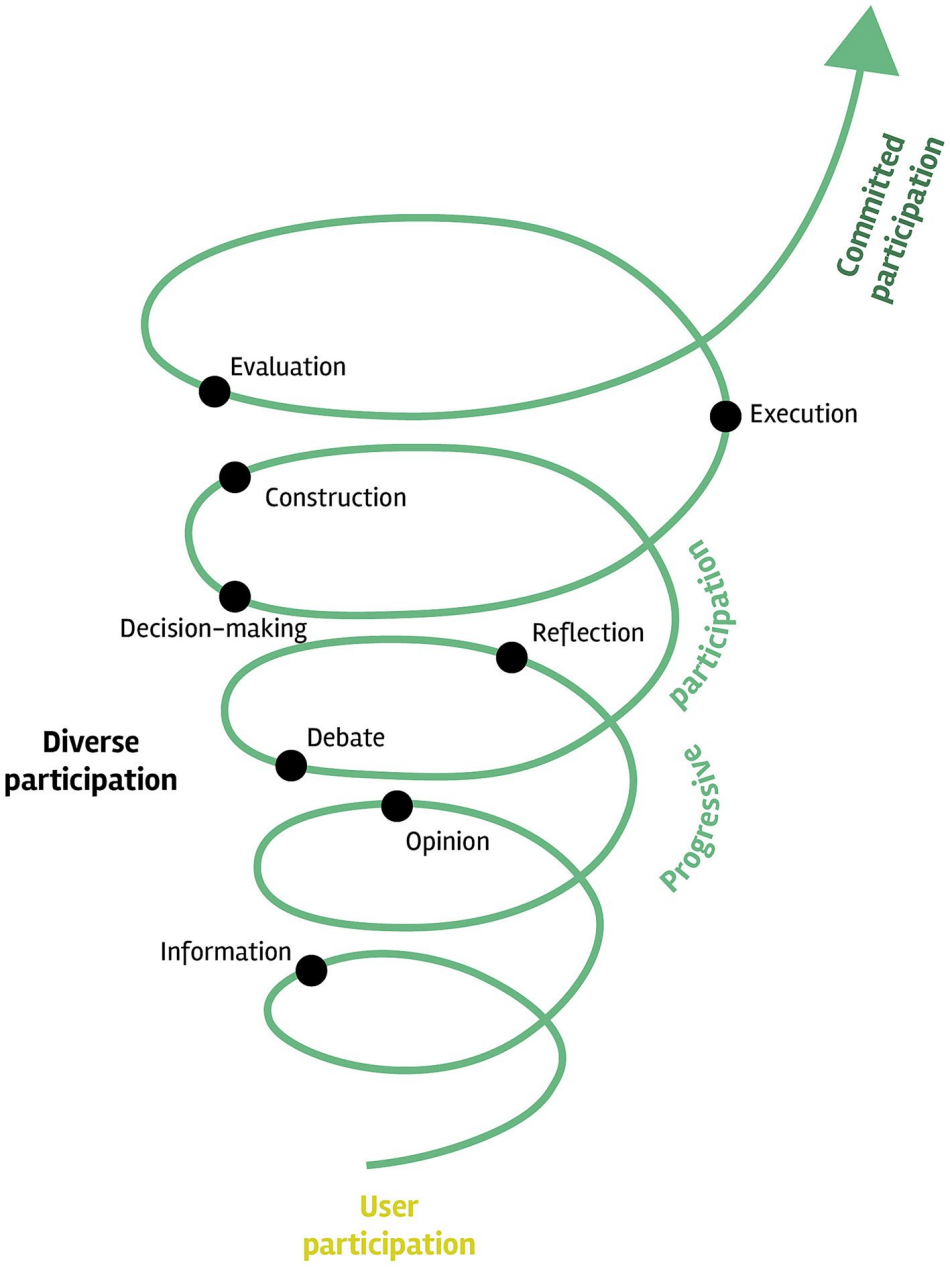
Spiral of participation.

As shown in Figure 4, rather than ascending a ladder or progressing from one rung to the next, the participatory process is conceived here as a spiral that begins when contact with the initiative is first established—whether by attending a workshop or an event—and from there unfolds in a spiraling fashion toward progressively deeper forms of participation. This metaphorical choice is not merely aesthetic: it aligns with critiques of the linear and hierarchical representation of participation, which can misleadingly suggest that occupying a higher rung on the ladder automatically entails greater citizen influence (Koomson, 2024; Rosen and Painter, 2019). As Rosen and Painter (2019) argue, even when communities formally reach the rung of citizen control, the economic, political, and cognitive asymmetries that shape local contexts may constrain their actual capacity to decide and implement. In this sense, the ladder model may be insufficient to account for the effective dynamics of power. From this critical standpoint, co-production is better understood not as a fixed endpoint, but as an iterative and dynamic process that requires the ongoing dismantling of barriers to meaningful engagement. In a complementary direction, Koomson (2024) proposes a bidirectional understanding of participation, according to which full engagement requires both that external promoters cede spaces of power and that local participants occupy and activate those spaces; a dynamic that, like the spiral, may advance, stall, or even regress depending on contextual conditions.

Facilitating participatory experiences that nurture the desire to become involved and to contribute to collective transformation is essential. As stated at the outset of the article, participating means “to be part of and give shape to” (Montañés, 2020), or, in the formulation of Rebellato and Giménez (1997), forming part of a group, having a stake in it, and taking part, which entails acting to transform. Participation refers to the process by which a community or organization deliberately assumes an active role in the construction of a shared future (Gorostidi and Martínez, 2023). From this perspective, it constitutes a practice that is simultaneously personal and collective, and one that is—or aspires to be—formative, educational, creative, egalitarian, and transformative (Ahedo, 2022). These understandings are consistent with the discursive position of committed participation identified in this research. Participation entails commitment, decision-making, a sense of belonging, and action oriented toward its realization. As discussed above, this involves dedication, responsibility, the capacity to decide, and the will to act for the collective well-being. Understood in these terms, committed participation is both ideological and political. Within this discursive position, all of these elements are regarded as essential for participation to exist.

The opposing position, identified as user participation, refers to being present or taking part in an activity or group in pursuit of personal benefit. Although these two conceptions of participation are, in principle, antagonistic, both seek to address needs, in line with the positions advanced by the authors reviewed above. In committed participation, the aim is the common good, which links it to participation understood as a need in itself, as described by Díaz Bordenave (1989) and Max-Neef et al. (1986). In user participation, by contrast, the needs addressed are diverse. As this research has shown, such needs may be explicit and evident, may emerge suddenly, or may remain latent as underlying needs. The coexistence of these plural motivations reinforces the idea that participation cannot be measured by a single standard: what constitutes a meaningful experience for one person, including the simple act of breaking out of isolation or reclaiming a public space, may be irrelevant or insufficient for another.

Faced with these two dichotomous options—one that posits essential elements for a process to qualify as participatory and another that could be summarized as “everything is participation”—this research identified two further positions that warrant consideration in the analysis of participatory processes, insofar as they bring additional perspectives into view. Diverse participation acknowledges that there are multiple ways of participating, without ruling out attendance as one such form. This mode of participation is synergistic, since some of its forms make it possible to address a personal need while simultaneously attending to collective ones, thereby contributing to the common good. Although these forms vary according to context, it is analytically useful to examine which levels are activated in any given participatory process: information, opinion, debate, reflection, collective construction, decision-making, implementation, and evaluation. The eight distinct steps that emerge from the discursive positions reconstructed in this study contrast with the tendency documented in other studies, where action plans derived from evaluation tools tend to concentrate disproportionately on the initial steps—information and communication—while neglecting the more structural ones, such as shared evaluation or the collective construction of knowledge (López-Ruiz et al., 2024).

Movement from one step to another corresponds to progressive participation, through which committed participation may be reached starting from user participation. This process involves traversing intermediate steps, akin to what Sirvent (1984) terms “semi-participatory steps.” The trajectory, however, is understood not as linear but as a spiral: there is no single possible trajectory. For this reason, the participatory process is represented as a spiral rather than a ladder. The spiral suggests a participatory journey that expands and opens up new possibilities as the process deepens. The trajectory is not governed by hierarchically ordered levels detached from one another; rather, it is built from steps that feed back into one another and that are not necessarily given in the same order. Not all people take the same steps, nor do they follow the same sequence or move through them at the same pace. It is possible to skip steps, or to experience some first and others later, to move forward and back, to pause or accelerate the rhythm. Likewise, what appears as a point of arrival may become the beginning of a new cycle. All of these are possible variations within the diverse participatory trajectories. This recursive quality is consistent with approaches in co-production theory, which understand participation not as a closed sequence but an iterative learning process in which each cycle reveals new obstacles and opportunities, thereby requiring the redefinition of previously adopted strategies (Rosen and Painter, 2019).

The direction of the spiral’s arrow, as shown in Figure 4, points outward rather than inward, driven by a centrifugal force that enables transformations with an impact on the wider community (Encina et al., 2018). The unfolding of the spiral requires the desire—linked, as noted, to needs—together with the knowledge and capacity to participate (Montañés, 2022), and entails a dialogic educational process centered on the development of critical consciousness (Freire, 2016), understood as the capacity to reflect on the foundations upon which each person’s reality is constructed (Villasante et al., 2012). This emphasis on critical reflexivity concerns not only participants but also those who facilitate the processes, who must examine their own positions, assumptions, and affects within the power relations that traverse the community space, rather than presenting themselves as neutral mediators (Alzate, 2020).

What is referred to here as user participation is not usually identified explicitly—neither by that term nor by any other—in the participatory cycles documented in the literature reviewed. Even so, this research suggests that such a starting point deserves particular attention. For some individuals, engaging in a collective process can be profoundly transformative, especially when they have been excluded from multiple spaces on the basis of gender, social class, cultural belonging, or other social markers, as reflected in the discourses produced across the conversational devices for this study, some of which is reproduced below:

I am thinking of specific cases of women [for whom] being part of a group has meant a dramatic change in their life history, from never having been able to leave the domestic sphere or the sphere of work… to becoming part of a group […] We cannot approach participation through a single variable, because it is surrounded by many other variables, which may be communal or personal […] Perhaps, for me, the fact that this woman comes to the group and sits there is not participation, and yet, in terms of her life history, it is fundamental […] We sometimes think about this [participation] at the community level, but we cannot stop looking at it through the lens of individual trajectories as well. (CD.1)

This account illustrates what recent literature has begun to conceptualize as the need to attend to “participations” in the plural: forms of engagement that coexist within the same space, that do not always conform to institutional definitions of what counts as participation, and yet produce real effects on individual trajectories and on the collective fabric (Den Dulk and Buizer, 2026). Recognizing their value helps prevent the emphasis on committed participation from becoming, paradoxically, a new form of exclusion toward those who, for diverse reasons, follow this path at a different pace or in a different direction. Attending to the dual trajectory of participatory processes is therefore essential. Doing so requires analyzing the paradigm from which participation is conceived and implemented, and the trajectories of the individuals themselves who navigate the participatory journey (Quintar et al., 2009). A double perspective must therefore be maintained: one focused on individuals and their personal trajectories, and another on the participatory process itself and its collective trajectory. This dual focus aligns with findings from other studies, in which the gap between declared and actual participation is explained, among other factors, by the difficulty of sustaining long-term individual accompaniment within initiatives that prioritize aggregate indicators of attendance or coverage (Cassetti et al., 2023).

A final caution concerns the risk of assuming that committed participation is inherently positive. Collective participatory processes therefore require sustained reflection on the ideology underlying each initiative or movement. As Cerletti (2008) observes, the positive valuation of participation tends to unify meanings where a diversity of interests may in fact exist. In the author’s words “the underlying assumption regarding the ‘common good’ obscures (renders invisible and delegitimizes) the possible variety of positions and constructions” (Cerletti, 2008, p. 8). Just as the meaning of participation should not be taken for granted, neither should the meaning of the common good. This caution is especially pertinent in light of recent findings showing how, even in contexts institutionally committed to participation, participatory practice can drift toward rhetorical or procedural forms that, unintentionally, reproduce preexisting asymmetries (Mariani et al., 2025). In this line, the proposal by Cassetti et al. (2023) to revisit the very language used to name participatory practices—distinguishing with precision among attending, consulting, and engaging—may be read as a methodological invitation to prevent the mere invocation of the word “participation” from functioning as a veil over the different intensities and asymmetries that coexist within any given process.

## 6 Conclusion

Research on SACUDE leads, in sum, to the conclusion that conceptions of participation are organized not around a binary opposition or a linear hierarchy but around a spiral linking four mutually interdependent positions—committed, user, diverse, and progressive. The principal contribution of this study lies in showing that these positions are not mutually exclusive alternatives, but complementary dimensions whose effective articulation demands differentiated and sustained strategies. In doing so, the manuscript contributes to the study of participation in community initiatives by offering a conceptual and analytical framework that accounts for the coexistence, tension, and mutual implication of different participatory positions within the same process.

The spiral offers a more faithful metaphor than the ladder for representing actual participatory processes, because it allows setbacks, bifurcations, and simultaneities; because it recognizes that each step—informing, expressing opinions, reflecting, debating, planning, deciding, implementing, and evaluating—can be both an endpoint and a point of departure; and because it assumes that the force of participation is projected outward only when the individual trajectories sustaining it are attended to at the same time.

For SACUDE and analogous initiatives, one remaining challenge is the development of evaluation tools capable of observing both trajectories, the individual and the collective, simultaneously, without diminishing the richness of either, together with the creation of spaces for critical reflection in which ideological assumptions about the “common good” can be named, discussed, and, where necessary, reformulated.

## Data Availability

The raw data supporting the conclusions of this article will be made available by the authors, without undue reservation.
